# Making decisions about antidepressant use during pregnancy: a qualitative interview study of a sample of women in the UK

**DOI:** 10.3399/BJGP.2024.0068

**Published:** 2025-03-25

**Authors:** Heather James, Sophie Smith, Dheeraj Rai, Iryna Culpin, Katrina Turner

**Affiliations:** Centre for Academic Mental Health, Population Health Sciences, Bristol Medical School, University of Bristol, Bristol.; Centre for Academic Mental Health, Population Health Sciences, Bristol Medical School, University of Bristol, Bristol.; Centre for Academic Mental Health, Population Health Sciences, Bristol Medical School, University of Bristol, Bristol.; Florence Nightingale Faculty of Nursing, Midwifery and Palliative Care, King’s College London, London; honorary research fellow, Centre for Academic Mental Health, Population Health Sciences, Bristol Medical School, University of Bristol, Bristol.; Centre for Academic Mental Health; Centre for Academic Primary Care, Population Health Sciences, Bristol Medical School, University of Bristol, Bristol.

**Keywords:** antenatal depression, antidepressants, decision making, medication, perinatal mental health, pregnancy

## Abstract

**Background:**

An increasing number of pregnant women now take antidepressants. Many pregnant women experience decisional conflict when deciding whether to take antidepressants, but little is known about the attitudes and experiences that influence these decisions.

**Aim:**

To explore the attitudes and experiences influencing women’s decisions about antenatal antidepressant use.

**Design and setting:**

A qualitative study using in-depth interviews with a sample of UK women who experienced antenatal depression or took antidepressants antenatally within the preceding 3 years.

**Method:**

Recruitment adverts were placed by a perinatal mental health charity and on parenting forums and social media platforms, resulting in a convenience sample. Interview data were coded and analysed with thematic analysis using QSR NVivo.

**Results:**

Twenty-two women were interviewed; one-half had taken antidepressants during pregnancy. Most women had concerns about adverse effects and viewed antidepressants as adjunctive to non-pharmacological treatments, which were reported as difficult to access. Some women reported that professional advice was insufficiently detailed. Women described the need to cope with their symptoms, their baby, and existing responsibilities, and related their decisions to their perceived ability to cope. This perception was influenced by physical and emotional challenges relating to pregnancy. Women’s decisions were influenced by their previous experiences and by the perceived societal expectations placed on pregnant women.

**Conclusion:**

Decision making is a complex and dynamic process, personal to each woman’s circumstances. Perceived ability to cope is an important factor in decision making. Detailed information should be offered to women for support with decision making in relation to antenatal medication use.

## Introduction

Following an increase in antenatal antidepressant prescribing over recent decades,^1^,^2^ almost 8% of women are prescribed antidepressants during pregnancy.^2^ Antidepressant discontinuation is more common in pregnant than non-pregnant women,^1^ likely related to concerns from women and/or health professionals about the risks of antenatal antidepressant use. Clinical guidelines recommend that pregnant women should be screened for mental health problems at their first contact with primary care or antenatal booking appointment.^3^ Women with mild to moderate perinatal depression should be managed within primary care and those with severe illness referred to a specialist perinatal mental health service.^3^,^4^ A substantial burden of perinatal mental illness is managed by GPs.^5^,^6^ GPs’ prescribing confidence for perinatal depression varies, with some GPs reporting low levels of confidence in providing information and a reliance on personal experience.^7^

The literature on risks is still inconclusive,^8^ but pregnant women consistently overestimate the estimated risks of antenatal exposure to antidepressants.^9^–^11^ However, continuing antidepressants during pregnancy may reduce the risk of relapse^12^ and reduce depressive symptom burden postnatally.^13^

Most previous studies on women’s attitudes to antenatal antidepressant use have utilised electronic surveys or questionnaires, producing data with limited depth.^14^ Additionally, previous literature has focused on perceived risks, with very few studies investigating perceived benefits.^14^ Pregnant women are commonly concerned about the risks of fetal and maternal adverse effects.^15^–^17^ Most women report seeking advice about treatment from healthcare professionals and friends and family;^16^,^18^ in one study the most influential advice came from GPs.^16^

**How this fits in**

Previous research on decision making about antenatal antidepressant use has lacked depth, focused on the concept of decisional conflict, and tended to include only women who decided to take antidepressants. This study used in-depth interviews with 22 women (including those who decided not to take antidepressants) to explore the attitudes and experiences driving these often complex decisions. We found that women’s decision-making processes were usually influenced by their personal perceptions of the perceived benefits and risks of antidepressants, previous experiences, current level of coping, and perceptions of societal expectations placed on pregnant women. Our results emphasise the need for professionals to apply a holistic approach when supporting women with these decisions and to actively enquire about the effects of physical and emotional wellbeing on perceived coping. There is also a need for better links between primary care and specialist perinatal services to support GPs with prescribing decisions in pregnancy.

A small number of existing qualitative interview studies have mostly focused on exploring the high levels of decisional conflict (difficulty making a decision, often resulting in emotional distress) reported by women making these decisions.^19^,^20^ A Norwegian interview study explored pregnant women’s attitudes and the decision-making process in more detail, and concluded that women’s decisions were often influenced by their perceived ability to parent and take part in family life. However, the study was small (*n* = 8) and included only a small number of women who discontinued (and did not re- start) antidepressants during pregnancy.^21^

The aim of this study was to explore the attitudes of UK women with a range of experiences (including both those who decided to take, and not to take, antidepressants) towards antenatal antidepressant use. The study was designed to move away from women’s descriptions of decisional conflict, to focus on the details of the attitudes and experiences driving these decisions. It is hoped that further knowledge in this area will enhance healthcare professionals’ skills and confidence in shared decision making for antenatal antidepressant use.

## Method

This was a qualitative study designed from an interpretivist perspective, which holds that a person’s experience of their own reality is individual, subjective, and socially constructed.^22^ Thus data were gathered through in-depth interviews that would allow participants’ views and experiences to be explored in detail, and then analysed using an inductive approach that maintained the focus on women’s interpretations and experiences. Ethical approval was granted from the University of Bristol Faculty of Medicine and Dentistry Committee for Research Ethics, and the study received input from the CEO of Mothers for Mothers, a Bristol-based charity that supports women with perinatal depression.

### Recruitment and sampling

Women were eligible for inclusion if they reported experiencing depression during pregnancy, and/or were taking antidepressants prior to conception. Inclusion criteria were intentionally broad to capture a wide range of women’s experiences. To minimise the potential for recall bias, a time limit of 1 year between the end of the pregnancy and the date of the interview was set. However, to improve recruitment volume and inclusivity, this was later increased to 3 years. Women were eligible for inclusion regardless of the outcome of their pregnancy.

Women who were currently pregnant or deciding what treatment to take for depression, at the time of interview, were excluded. This was to prevent women’s decisions from being influenced by participation in the study.

An advert was circulated to members of Mothers for Mothers, on social media (via relevant organisations such as perinatal depression charities), and on mental health forums of parenting websites. This resulted in recruitment via convenience sampling, and it was hoped that using widely and publicly available web-based platforms would achieve demographic diversity within the sample. The advert stated that the research team wanted to hear from women who had experienced depression during pregnancy and were willing to take part in an interview to discuss the decision to take or not take antidepressants. Advert responders were sent further information including eligibility criteria and a participant information leaflet. Those who were eligible and agreed to take part completed a written consent form via post or email attachment. Some recruitment also occurred via snowball sampling by word of mouth. Towards the end of the recruitment and interviewing process, purposive sampling was used to preferentially recruit women who decided not to take antidepressants, to ensure their experiences were explored as fully as those who decided to take antidepressants.

Participants received a £10 gift card to thank them for taking part. Recruitment stopped when data saturation had been reached, which was deemed to have occurred when no new themes were identified in three consecutive interviews.

### Data collection

Participants were given a choice of being interviewed in person, by telephone, or by video call. The interviews were conducted by the first author, a female academic foundation doctor with an interest in perinatal mental health, experience in interviewing, and training in qualitative research methods. A topic guide was used to ensure consistency across the interviews (Supplementary Information S1). It was informed by relevant literature and included questions designed to elicit information needed to address the aim of the study and focused on participants’ pregnancy journey (including their mental health), views about treatment options, sources of advice and support, and how they reached a decision about antidepressant use.

Prior to the interview, participants also completed a socioeconomic questionnaire so that the characteristics of women interviewed were known.

### Analysis

Drawing on the principles of grounded theory,^23^ data collection and analysis were done in parallel so that initial insights could inform the focus of later interviews and to identify when data saturation had been reached. Interviews were transcribed verbatim, anonymised, and analysed thematically.^24^ Interview transcripts were read and re-read (by the first author) to facilitate familiarisation with the data. An initial subset of transcripts were coded independently by two researchers (the first and fourth authors), and a coding framework agreed. The first and second authors independently coded a further subset of data using this framework, and then met to compare their coding and interpretation of the data. Any discrepancies in coding were addressed through discussion and the coding frame was revised slightly to ensure its completeness and to avoid codes overlapping. Having finalised the coding frame, it was then applied to all the transcripts. The data were coded in NVivo 11 to facilitate data management. Once all the transcripts had been coded, using an approach based on framework analysis,^25^ data under specific codes were retrieved and summarised in tables, where rows represented interviewees and the columns specific codes. Having summarised the data, the researchers compared findings within and across the interviews to identify key themes and deviant cases. Data collection and analysis were overseen by the fifth author, a Professor of Primary Care Research and expert qualitative methodologist.

## Results

Twenty-two participants were interviewed between December 2018 and August 2019: three face-to-face and 19 by telephone (Table 1). The duration of interviews ranged from 29–70 minutes.

**Table 1:** Socioeconomic characteristics of interview participants

Total *N* = 22	Number	%	Standard deviation
Mean age (years)	32.3		5.9
Mean age of last child (months)	16.4		9.0

**Educational qualifications**			
No or below degree-level qualifications	6	27%	
Degree/HND/NVQ Level 4 or above	16	73%	

**Present employment situation**			
Working full- or part-time (paid)	16	73%	
Unemployed and looking for a job	1	5%	
Other	5	23%	

**Partner**			
Yes	19	86%	
No	3	14%	

**Ethnicity**			
White British	20	91%	
Other	2	9%	

HND = Higher National Diploma. NVQ = National Vocational Qualification.

One-half (*n* = 11) of the participants reported taking antidepressants at some point during their pregnancies. Women consulted various professionals with their depressive symptoms: 15 consulted their GP, six consulted their midwife, five consulted specialist mental health professionals including psychiatrists, four consulted an obstetrician, two consulted a mental health midwife, and one consulted an IVF consultant. Three women did not seek any professional help at all.

The sample appeared to represent women with a range of illness severity: one woman underwent a psychiatric admission during her pregnancy, four were referred to a specialist perinatal mental health team by their midwife or GP, and the remaining 14 women who sought professional support were managed within community midwifery services or primary care.

Eight women decided to continue or initiate antidepressants during pregnancy, three women initially stopped antidepressants before or early in pregnancy and then re-started in the second or third trimester, and 11 stopped taking antidepressants during pregnancy (or decided not to initiate them during pregnancy).

The results of the thematic analysis are presented below. The main themes identified were ‘Information seeking and advice’ and ‘Attitudes to antidepressants’. Within the second theme, two subthemes were identified: ‘The ability to cope’ and ‘Concerns about antidepressant use’. Quotes have been reproduced to illustrate points made and tagged using a unique identifier.

### Theme 1: Information seeking and advice

Most women reported discussing treatment options with their friends and family, as well as with professionals. Many women described receiving information from professionals that was balanced, involving discussion about possible risks and benefits. However, P5 described feeling pressurised into taking antidepressants by her psychiatrist during an inpatient admission due to the severity of her illness, and P13 said her obstetrician had encouraged her to discontinue antidepressants because of severe sickness and a drug interaction with her antiemetic. Although some women were happy with the advice they had received, others said they would have preferred more detailed information about specific risks and benefits. Several women referenced a lack of scientific evidence about antenatal antidepressant use resulting in uncertainty:

*‘You know, no one really does trials on pregnant women so — I remember that massive caveat, no one does trials on pregnant women so we don’t really know, it’s like we think it’s alright but we’re not sure.’* (P2)

Several women said they felt reassured after receiving advice about the specific antidepressant they were taking. Four women received reassuring advice from their GPs, two from a consultant (fertility specialist and psychiatrist), and one from a perinatal mental health nurse:

*‘I just remember the emphasis on the risk of* [sertraline] *being the safest one when I was pregnant.’* (P20)

Although most women said that their decision had not been influenced by social media or websites (which they viewed as unreliable), some women reported doing their own research on the internet. P10 reported that she had stopped taking antidepressants after reading on an online parenting forum about a miscarriage that was reported to have resulted from antidepressant use. Five women, who all had science or publishing backgrounds, described the importance of consulting reliable sources, including scientific literature, to inform decision making.

Women indicated that professional advice was perceived as particularly reliable when the professional had a specialist interest in both pregnancy and mental health. Interview participants generally valued the advice and support from GPs, but some women felt their GP lacked specialist knowledge about pregnancy or mental health:

*‘I think your first port of call is your GP and they can’t be an expert on everything so maybe they don’t really have the knowledge or the information they can give to you about medication.’* (P7)

Three women reported not seeking any professional mental health support during their pregnancies and did not take antidepressants. These women described a fear of approaching professionals for a variety of reasons: P6 had been advised by her mother (a nurse) that this could result in removal of her baby; P6 and P9 were afraid they might appear ‘a failure’; P9 was worried she might be put under pressure to take antidepressants; and P11 lacked confidence following a negative experience when previously seeking help for low mood. Two of these women also described feeling that antenatal and postnatal appointments appeared to lack focus on the mother’s wellbeing:

*‘*[At antenatal appointments] *It was always very much check on the baby, check on the baby, is the baby OK and then it just kind of felt like once that was all done that was it … it never seemed to crop up that we were both important in that.’* (P9)

*‘I’d mention it to* [health visitor on postnatal visit] *that I don’t feel like going out, I don’t feel happy, she would say OK and like — I said it about five, six times and then like — something tripped and she was like oh maybe you should speak to your GP … it was dismissed quite a few times by the health visitor.’* (P11)

### Theme 2: Attitudes towards antidepressants

#### The ability to cope

Many women who ultimately decided to take antidepressants during pregnancy described high symptom burden and struggling to look after themselves, and in turn their babies, when making their decisions:

*‘When I’m not on my medication I have … well I call them episodes … where I’m very anxious or the other end I’m very depressed, I can’t really function very well on a day-to-day basis and obviously that’s not — you’re not in the best place to look after yourself let alone if you’ve got a baby.’* (P3)

Many women described challenges that were related to being pregnant, including difficult emotional responses (negative feelings towards the pregnancy, anxiety about fetal wellbeing) and physical stressors (nausea and vomiting, tiredness, physical discomfort). These caused additional stress on top of existing challenges, including those related to work, relationships, and caring for existing children, leading to a reduced sense of coping:

*‘So moving to a new* [work] *site with a terribly sick teacher who can’t get a decent night’s sleep, whose daughter isn’t sleeping a hundred per cent all the time anyway … you can imagine it was a toxic combination, absolutely toxic … it got to the point in the January I ended up in floods of tears ’cos I was just being sick most days.’* (P4)

In some cases, the trigger for starting antidepressants during pregnancy was a crisis, for example, a strong negative reaction to a gender scan or experiencing suicidal ideation:

*‘I said “this isn’t normal, I’m a mum, I shouldn’t feel this thing that I’m feeling. I feel awful and evil and I should be caring and nurturing and yet I just want him gone” … then I thought do you know what, I’m just going to crash my car, it’ll be easy … So I braked at the last moment, missed by, don’t know, wholly surprised I missed it but I did and then the doctor — I went and saw the GP, they put me on sertraline.’* (P12)

Three women who initially stopped their antidepressants shortly before or in the early stages of pregnancy said their depressive symptoms re-emerged in the second or third trimester:

*‘A tidal wave on the horizon.’* (P2)

They attributed this resurgence of symptoms to the discontinuation of antidepressants at a time of increased physical or emotional stress and decided to re-start their antidepressants.

Some women believed that antenatal antidepressant treatment might mitigate the risk of developing postnatal depression. This was particularly the case for women who had suffered previously from postnatal depression, often with distressing and disruptive sequelae:

*‘It was a really big fallout if I became really, really unwell, you know, the impact on my other children … With my first baby I was in hospital for three months with her … if I had to go into hospital again with my other babies I’m leaving my other children behind … missing out on them … they’re missing out and a big horrible, ugly mess basically.’* (P7)

Other women described coping well while taking their antidepressant and not wanting to risk disruption resulting from discontinuation. In some cases, any potential adverse effects of antidepressants were perceived to be outweighed by the possible effect of depression itself. Conversely, some women who described coping well with their symptoms and day-to-day life felt that the potential risks outweighed the benefits and opted to stop their antidepressants in anticipation of, or shortly following, conception:

*‘Things had been fine for quite a long time so I wasn’t particularly anxious and I wasn’t experiencing a great deal of stress at the time … I just decided* [continuing antidepressants] *wasn’t a risk that I needed to take.’* (P17)

#### Concerns about antidepressant use

Almost all women in the sample expressed concerns about the risk of adverse fetal effects. These were sometimes non-specific, but many women did cite specific concerns including effects on brain chemistry, risk of cardiac abnormalities, neonatal addiction/withdrawal, and neonatal respiratory distress. Two women who decided not to take antidepressants both had a previous history of miscarriage and expressed concerns about possible pregnancy loss. For one woman, this concern prevented her from seeking professional help:

*‘’Cos of the history of miscarriage and because of this whole I’m so lucky to be pregnant, I didn’t want to do anything that would compromise it … I think as well that was a factor of me not admitting my mental health problems because I think they would have prompted me to have treatment and in my head that would have been straightaway medication and I know I wouldn’t have taken it.’* (P9)

Only one woman described having no concerns about fetal wellbeing. She reported feeling emotionally detached from her pregnancy. Her concerns about taking antidepressants were centred more around the potential for her to experience adverse effects, and about the potential effects on her existing child.

Some women considered that safety in breastfeeding was also an important factor, especially those who had had previous (positive or negative) experience of breastfeeding.

A number of women described their intolerance to fetal risks, even when they perceived them as minimal. These women also described high levels of anxiety relating to fetal health, fear of experiencing guilt, and anticipation of self-blame in the event of an adverse outcome:

*‘I think if he ended up having any, I don’t know, having difficulties or even mental health problems himself, I didn’t want to feel like I might have contributed to it by doing something during pregnancy.’* (P19)

Many women referenced the idea of the sanctity of the pregnant body, the reluctance to introduce ‘unnatural’ substances, and a preoccupation with healthy living. Some women described a sense of fear and uncertainty about taking any medication at all during pregnancy.

*‘I didn’t take paracetamol, I didn’t take ibuprofen, I didn’t really take any kind of drugs when I was pregnant. I struggled coming to terms with getting — to having Gaviscon with my heartburn (laughs).’* (P7)

Several women described making their decisions within the context of the perceived societal expectations placed on pregnant women: to feel joyful, and to nurture the pregnant body. This was described by P17 as the ‘*surveillance*’ of pregnant women. Women described the stigma associated with being mentally unwell or taking antidepressants during pregnancy:

*‘I just felt that if I said “oh I’m on medication for my mind”, that might be seen as a selfish thing to do, you know, the equivalent I guess of maybe tucking away a bottle of wine a night or something by people.’* (P2, who initially stopped her antidepressant and then re-started during pregnancy)

Two women (P9 and P18) who worked in the mental health field described feeling particularly ashamed and vulnerable to criticism. Both women decided not to take antidepressants.

For some women, the desire to avoid medication during pregnancy was rooted in the context of their personal experiences. Following an overdose early in her pregnancy, P5 reported feelings of guilt and tried to avoid any medication for the rest of her pregnancy, but later re-started her antidepressants during an inpatient psychiatric admission. P10 reported that her drink was ‘spiked’ shortly before becoming pregnant, leading her to fear taking any medication. Another woman described a neighbour with mental health problems as someone she feared she would become like if she took antidepressants:

*‘I was just so frightened of becoming like her, so dependent on* [antidepressants] *and if she’s running low towards the end of her pack she gets jittery and “oh my God, is this normal?” It’s like she’s a drug addict.’* (P12)

Due to their concerns about taking antidepressants during pregnancy, most women said they preferred to try non- pharmacological strategies before, or instead of, antidepressants. Many women described antidepressants as an adjunct to non-pharmacological strategies:

*‘A buffer.’* (P7)

*‘A hand to hold … a boost up.’* (P10)

*‘A crutch.’* (P2)

Most women reported seeking non-pharmacological strategies: most commonly counselling (often private), help from charities or local support groups, and exercise. However, women reported a range of barriers to accessing these strategies, including long waiting lists for talking therapies and difficulty accessing perinatal mental health services. Such barriers, alongside the time-limited nature of pregnancy itself, contributed to some women’s decisions to start antidepressants:

*‘*[My GP] *said by the time they get referred to you, you will have had the little one so she said you might need something before that ’cos you’re not coping … it did make sense and I appreciated her saying it so I could get treatment there and then.’* (P15)

## Discussion

### Summary

When making their decisions, most women sought advice and support from friends, family, and professionals. The majority had consulted their GP. Many women wished they had received more detailed information about the risks and benefits of antidepressants. Professional advice was perceived as most valuable when it came from someone with specialist interests in both pregnancy and mental health.

Most women were able to identify both potential advantages and disadvantages of taking antidepressants, regardless of their decisions. The unique emotional and physical effects of pregnancy were described by many women as affecting their ability to cope with existing stressors, which in some cases directly impacted their decisions about antidepressants.

Nearly all women expressed concerns about fetal risks, and perception of these risks was the main reason cited for not taking antidepressants. Some women described high levels of anxiety about fetal health, along with concerns about experiencing guilt and self-blame in the event of an adverse outcome. Women described decision making as a dynamic process, in the context of their previous experiences and their current (often evolving) psychosocial situations. Women’s decisions were also influenced by the availability and perceived usefulness of non-pharmacological strategies.

Many women identified with the concept of the societal expectations placed on pregnant women, and described a stigma associated with experiencing mental health difficulties, or taking antidepressants, during pregnancy.

### Strengths and limitations

The use of in-depth interviews allowed women to describe their views and experiences in detail, and to raise points that were important to them. The inclusion of women who both decided to take, and not to take, antidepressants during pregnancy allowed exploration of the attitudes and decision-making processes of women with a range of experiences.

However, their retrospective accounts were open to recall bias and influence by whether or not they had taken medication when pregnant. Diagnostic or clinical measures of depression were not available and women self-reported as having experienced depression.

Furthermore, the study used convenience sampling and those interviewed were predominantly White ethnicity and over one-half were educated to degree level or above. This may limit the generalisability of our findings, especially because Black and Asian women and socioeconomically disadvantaged women experience barriers to engaging with antenatal care 26–29 and socioeconomic deprivation and psychosocial stress are associated with perinatal depression.^6^,^30^

### Comparison with existing literature

Our results echo previous findings that women with antenatal depression prefer to explore non-pharmacological treatments, such as psychotherapy, before considering antidepressant treatment,^19^,^31^ but experience barriers to accessing or engaging in these.^31^ As in previous research,^16^,^18^,^19^ we found that most women sought advice from professionals and/or friends and family, and that women sought to discuss with people who would understand their situation.^21^ Our results align with previous findings that women are commonly concerned about the risk of adverse fetal effects.^15^–^17^,^19^ In line with previous literature,^19^,^20^ many women in our study expressed decisional uncertainty, partly owing to a perceived lack of good- quality detailed information. Women’s accounts of their difficulties accessing specialist services is consistent with the suggestion that, despite recent expansion of specialist perinatal services,^32^ current provision is still a ‘postcode lottery’.^33^

We found that women’s perceived ability to cope (with their symptoms, their baby, and other responsibilities including existing children and work) was an important influence on decision making. The need to cope was also a key finding in the Danish interview study,^21^ which characterised women’s decisions as an attempt to fulfil the discourse of being a ‘good mother’, whether or not they took antidepressants during pregnancy. Similarly, we found that women sought to balance the risks and benefits of antidepressants to make a decision that felt right in their individual circumstances.

Our results also highlight the physical and emotional stressors experienced by women during pregnancy. In a previous study, women with symptoms of depression and anxiety reported that pregnancy-related physical symptoms affected their mental wellbeing by reducing their ability to complete normal activities and engage with coping strategies, including exercise.^34^ Although it is known that physical symptoms (such as pain, nausea and vomiting, poor sleep) and emotional stressors (such as stress and anxiety) affect quality of life in pregnant women^35^ and are determinants of antenatal depression,^36^ women in our study described the impact that pregnancy-related stressors had on their perceived ability to cope and subsequent decisions about antenatal antidepressant use.

Women described stigma around mental illness in pregnancy and three women did not disclose their depressive symptoms to any health professional. In one UK survey, nearly one-third of women reported that they did not disclose symptoms of mental illness in pregnancy and only 11% of women recalled being screened at booking.^37^ This is consistent with our finding that some women felt appointments lacked focus on maternal wellbeing. The reasons the women in our sample gave for not disclosing echoed those given in the UK survey — primarily embarrassment, a fear that their baby would be removed from their care, or doubts about the usefulness of consulting a professional.^37^ Women’s unwillingness to disclose symptoms of poor mental health is an important finding considering the recent Mothers and Babies: Reducing Risk through Audits and Confidential Enquiries across the UK (MBRRACE-UK) enquiry into maternal deaths and morbidity, which identified suicide as the leading direct cause of death in women between 6 weeks and 12 months postnatally, and described postnatal care as poor and insufficiently joined up.^38^

### Implications for research and practice

GPs support women facing decisions about antenatal antidepressant use. They have an important role in preconception and early pregnancy counselling, particularly given the even higher levels of decisional conflict in women planning a pregnancy versus pregnant women.^39^ GPs should be aware of the many and diverse factors that might affect a pregnant woman’s decision, and that women may not disclose symptoms due to fear and the stigma that surrounds poor mental health. This highlights the importance of primary care professionals identifying signs of mental health difficulty in pregnant and postnatal women who could potentially fall through the gap.

Women concerned about taking antidepressants during pregnancy want to receive detailed information about the risks and benefits, but GPs might not have the requisite knowledge.^7^ GPs can access guidelines from local formularies, and evidence-based information from the UK Teratology Information Service (UKTIS; for professionals) and Best Use of Medicines in Pregnancy (BUMPS; for patients). However, there remains a need for improved links with perinatal services to ensure GPs are appropriately supported.

Perceived ability to cope is an important factor that influences women’s decisions, and professionals should enquire directly about pregnancy-related physical and emotional stressors that could be targeted to improve perceived coping.

Considering the limitations of this study, further research is warranted into decision making about antenatal antidepressant use by women from ethnic minority and lower socioeconomic status backgrounds, as their attitudes and experiences may differ from those described by our sample. Identifying and challenging differences in the way socioeconomically disadvantaged and ethnic minority women experience antenatal care is important given the disproportionately high maternal mortality rates in these groups, and the finding that nearly 40% of perinatal deaths are mental- health related, including suicide.^38^

## Supplementary Information



## Data Availability

Data available on request to the corresponding author.
