# Supplementary material for: Making decisions about antidepressant use during pregnancy: a qualitative interview study of a sample of women in the UK
Source: Br J Gen Pract. 2025 Mar 25;75(755):e440–7. doi: 10.3399/BJGP.2024.0068 (PMC11966528; doi:10.3399/BJGP.2024.0068)
Supplement: Supplementary file 1 [file BJGP.2024.0068_suppl.pdf]

Supplementary Information S1

Interview Topic Guide

1. **To start with, can you tell me a bit about yourself and your family?**
   (Prompts: how many children, how old is youngest child, partner, job)

2. **I'm interested in hearing about what happened during your (most recent) pregnancy. Sometimes it's helpful to talk in the order in which events happened. Can you remember when you became pregnant – how you were feeling and what was going on?**
   (Prompts: what was she doing/living, when did depression start, was she on/had taken antidepressants before, why was she feeling depressed, how had she felt during previous pregnancies and, if depressed, how had she coped)

3. **…And during your pregnancy, how did you feel mentally?**
   (Prompts: change in symptoms/development of symptoms, life situation, start to talk about treatment)

4. **What were your thoughts about treatment at that time? What were your views on the different options?**
   (Prompts: ensure cover antidepressants - ask about perception of risk/benefits to both her and baby)

5. **What were your biggest worries about being depressed during your pregnancy?**
   (Prompts: what threats did depression pose)

6. **How did you cope? What did you do? Did you seek any advice or support? Did anyone discuss with you treatment options for your depression when you were pregnant?**
   (Prompts: healthcare professionals, friends, family, partner, media, Internet)

7. [If treatment options were discussed] **Which treatment options were discussed with you? … What were you told, what information were you given? What experiences did you have of the treatments suggested? What did you think about these options? … What advice did you get from others?**
   (Prompts: concerns/perceived benefits of antidepressants, attitudes towards other treatments, advice from different sources)

8. [If has had previous pregnancies] **How were things in your previous pregnancy(ies)? How did you feel mentally back then?**
   (Prompts: experiences of depression/treatment during previous pregnancies)

9. **What views did your partner have about treatment options for depression during your pregnancy?**
   (Prompts: perceived level of support, partner's role, partner's opinions)

10. **How confident did you feel about the decision you made? What was it like for you?**
    (Prompts: adequacy of information, weighing up info/opinions from different sources, decisional conflict, experience of treatment/not having treatment)
